# Efficacy and safety of nanoparticle albumin-bound paclitaxel plus cisplatin or nedaplatin plus tegafur/gimeracil/oteracil as induction chemotherapy regimen for hypopharyngeal cancer

**DOI:** 10.3389/fonc.2025.1504658

**Published:** 2025-02-24

**Authors:** Cailing Jiang, Yulan Liu, Yunyan Mo, Lieyin Xu, Lin Zhu, Zhenya Li, Shengyuan Xu, Xi Qin, Guangteng Wu, Mafei Kang, Xiaosong He, Feng Xue

**Affiliations:** ^1^ Department of Oncology, Affiliated Hospital of Guilin Medical University, Guilin, Guangxi Zhuang Autonomous Region, China; ^2^ Department of Radiotherapy, Affiliated Hospital of Guilin Medical University, Guilin, Guangxi Zhuang Autonomous Region, China; ^3^ Department of Radiology, Affiliated Hospital of Guilin Medical University, Guilin, Guangxi Zhuang Autonomous Region, China; ^4^ Department of Otolaryngology, Affiliated Hospital of Guilin Medical University, Guilin, Guangxi Zhuang Autonomous Region, China

**Keywords:** hypopharyngeal cancer, induction chemotherapy, nanoparticle albumin-bound paclitaxel, tegafur/gimeracil/oteracil, progression-free survival

## Abstract

**Background:**

Treatment of hypopharyngeal carcinoma involves surgery, radiotherapy, and chemotherapy. The combination of docetaxel, cisplatin, and 5-fluorouracil as a standard induction chemotherapy regimen allows enhanced laryngeal preservation after surgery. In this study, our objective was to retrospectively analyze the short-term efficacy and adverse events of nab-paclitaxel plus cisplatin or nedaplatin plus tegafur/gimeracil/oteracil as an induction chemotherapy regimen for hypopharyngeal cancer.

**Methods:**

This retrospective study involved 19 patients who received nab-paclitaxel plus cisplatin/nedaplatin plus tegafur/gimeracil/oteracil every 21 days intervals for three cycles at the Affiliated Hospital of Guilin Medical University (December 2020 to February 2023). The primary endpoint was progression-free survival. Adverse events were assessed in all patients.

**Results:**

Treatment response was evaluated after the second cycle. Clinical outcomes indicated that 2 (10.53%), 15 (78.94%), and 2 (10.53%) patients exhibited clinical complete response, partial response, and stable disease, respectively. The objective response and disease control rates were 89.47% (17/19) and 100%, respectively. The pathological complete response rate was 71.43% (5/7) among the seven patients who underwent surgery after three cycles. Following induction chemotherapy, 4 (21.05%), 2 (10.53%), and 2 (10.53%) patients received radiotherapy, chemotherapy, and chemotherapy plus immunotherapy, respectively, whereas 4 (21.05%) patients discontinued treatment. At the 17.43-month median follow-up, median progression-free survival was 17.6 months (95% confidence interval, 13.9-21.2). The most common grade 3 treatment-related adverse events were alopecia (36.8%), leukopenia (26.3%), and anemia (15.8%). No grade 4/5 treatment-emergent adverse events were observed.

**Conclusions:**

The combination of nab-paclitaxel, cisplatin/nedaplatin, and tegafur/gimeracil/oteracil is a safe induction chemotherapy for treating hypopharyngeal cancer.

## 1 Introduction

Head and neck cancers rank among the top 10 cancers globally in terms of incidence (1, 2), with hypopharyngeal cancer constituting 3% - 5% of head and neck malignancies (3, 4). Hypopharyngeal cancer is usually classified into pyriform sinus cancer, posterior pharyngeal wall cancer, and postcricoid region cancer according to its primary anatomical site. The main clinical manifestations of patients with hypopharyngeal cancer include a foreign body sensation in the pharynx, cervical lymph node enlargement, and dysphagia. However, the exact cause of hypopharyngeal cancer remains unclear. Current research indicates that hypopharyngeal cancer may be related to various factors, such as smoking, alcohol consumption, Epstein - Barr virus infection, human papillomavirus infection, etc. (5). When compared with other head and neck tumors, hypopharyngeal cancer is deeply located and concealed. It exhibits no obvious early - stage symptoms. Moreover, between 60% and 80% of patients present with cervical lymph node metastasis at the time of initial diagnosis, which is one of the primary factors contributing to a poor prognosis (6). Surgery served as the primary modality for treating laryngeal cancer before the 1990s. Currently, the National Comprehensive Cancer Network (NCCN) guidelines advocate either surgery or radiation therapy as the recommended approach for early pharyngeal cancer (7).

The management of locally advanced hypopharyngeal carcinoma involves surgery, radiotherapy, and chemotherapy (8). However, the Veteran Affairs and European Organization for Research and Treatment of Cancer Trial 24891 (EORTC24891) has promoted the development of a combination of induction chemotherapy and radiation therapy as a viable option for treating locally advanced hypopharyngeal cancer (9, 10).

Currently, the standard induction chemotherapy involves a combination of docetaxel (T), cisplatin (P or DDP), and 5-fluorouracil (F), offering opportunities for enhanced laryngeal preservation post-surgery in select patients (7). A phase III study on 213 patients with untreated stage III or IV laryngeal or hypopharyngeal tumors demonstrated that induction chemotherapy with T+P+F (TPF) increased larynx preservation and larynx dysfunction-free survival compared to PF (11). However, the TPF regimen may cause serious adverse events (AEs), including leukopenia, neutropenia, anemia, thrombocytopenia, alopecia, diarrhea, vomiting, lethargy, renal insufficiency, ototoxicity, and peripheral neuropathy (12, 13). The reduction of AEs associated with the TPF regimen and the enhancement of its effectiveness during the induction phase are pivotal focus areas for developing novel treatments for hypopharyngeal cancer.

Considering the current literature emphasizing induction chemotherapy with traditional regimens for hypopharyngeal cancer treatment and the recognized challenges of AEs, we aimed to retrospectively analyze the short-term efficacy and AEs of an alternative induction chemotherapy approach, specifically, the combination of nab-paclitaxel (N-P) plus cisplatin or nedaplatin (NDP) plus tegafur/gimeracil/oteracil (S-1), to provide insights into its effectiveness as a potential option for hypopharyngeal cancer induction therapy.

## 2 Materials and methods

### 2.1 Patients

Twenty-four patients diagnosed with hypopharyngeal cancer at the Affiliated Hospital of Guilin Medical University between December 2020 and February 2023 were screened. The inclusion criteria were: 1) histologically and radiographically confirmed hypopharyngeal cancer; 2) first diagnosis of hypopharyngeal cancer in patients who had not previously received treatment; 3) a measurable lesion according to the Response Evaluation Criteria in Solid Tumors (RECIST) version 1.1 (14), complete magnetic resonance imaging (MRI), and computed tomography evaluation data before and after induction therapy; and 4) Eastern Cooperative Oncology Group performance status of 0 or 1. The exclusion criteria were as follows: 1) other primary tumors of the head and neck; 2) prior chemotherapy, radiotherapy, or surgery; and 3) severe comorbidities (such as significant cardiovascular disease, viral infections, or major psychiatric illness). Finally, 19 patients met the inclusion criteria and were included in this study. This retrospective study was approved by the Medical Ethics Committee of the Affiliated Hospital of the Guilin Medical University, and all study participants provided informed consent.

### 2.2 Treatment

**Figure 1:**
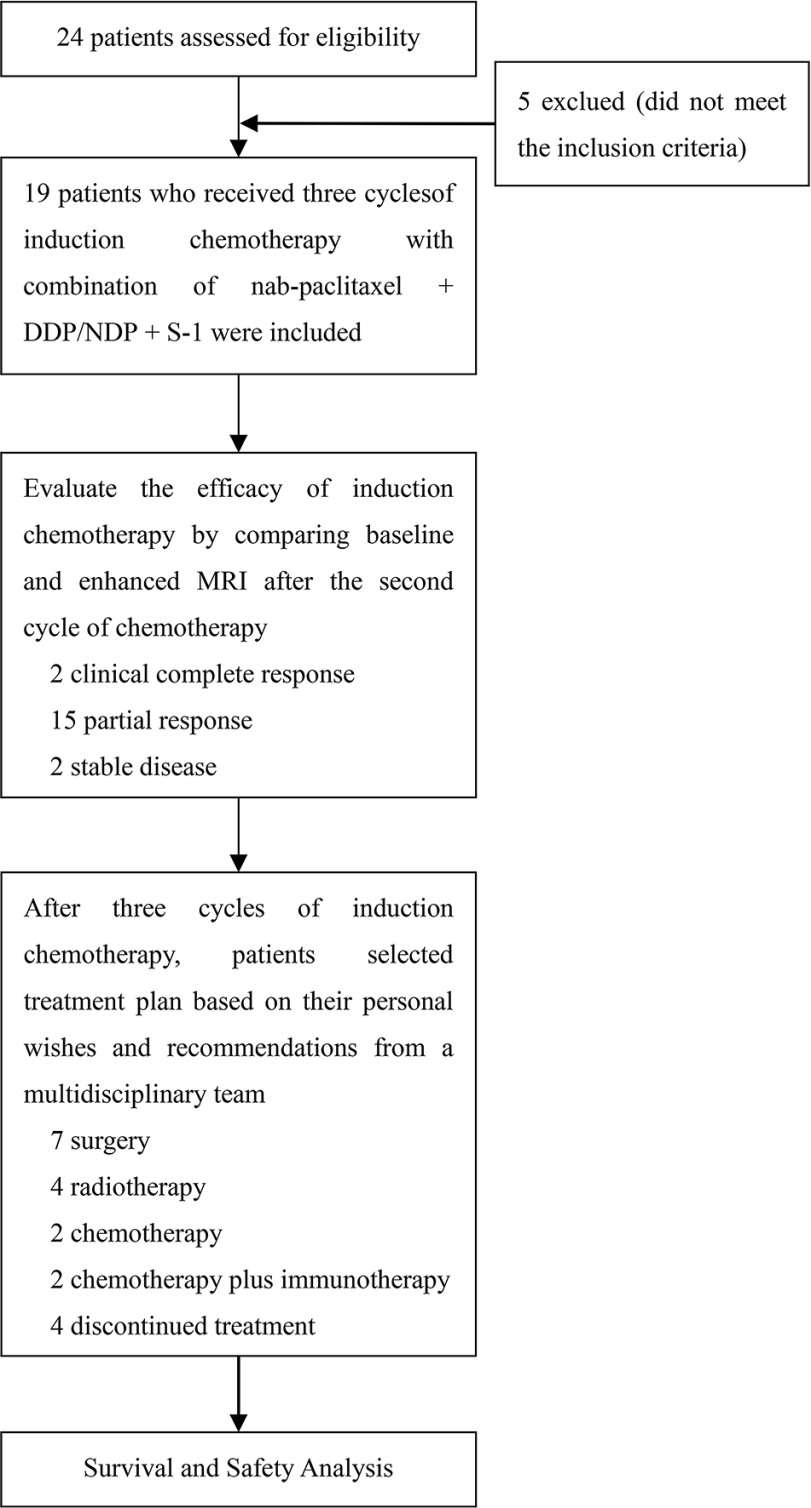
Study flow diagram.

In this study, 19 patients received N-P (125 mg/m^2^, days 1 and 8) plus DDP (35-40 mg/m^2^, days 1 and 2) or NDP (65-75 mg/m^2^, day 1) plus S-1 (30-40 mg/m^2^ twice daily [bid], days 1-14) every 21 d for three cycles. Baseline enhanced MRI of the head and neck was performed in all patients to confirm the presence of suspicious lesions. An additional MRI was performed before the third cycle of induction chemotherapy to assess efficacy. Subsequent treatment plans, such as surgery, radiotherapy, and chemotherapy, were chosen by the patients based on their preferences after a multidisciplinary team provided corresponding treatment suggestions following a comparison of the two MRI scans. The study design is illustrated in **Figure 1**. Patient responses were assessed using RECIST v1.1, and AEs were recorded throughout the treatment period and graded according to the Common Terminology Criteria for Adverse Events version 5.0.

### 2.3 Endpoints and assessment

The primary endpoint was progression-free survival (PFS), defined as the time from randomization to the first documentation of progressive disease per modified RECIST 1.1, according to the investigator’s assessment, or death from any cause (whichever occurred first). In cases where progression, relapse, or death did not occur before the cutoff date, the data were censored at the time of the last valid assessment preceding the cutoff date. Secondary endpoints included objective response rate (ORR) and disease control rate (DCR).

### 2.4 Statistical analysis

Data on patient demographics, tumor location and size, AEs, and survival were collected and analyzed. PFS was analyzed using the Kaplan–Meier method, with associated 95% confidence intervals (CIs) calculated using the Clopper–Pearson method. Statistical analyses were performed using Statistical Package for Social Sciences version 27.0 (IBM Corp., Armonk, NY, USA). Waterfall, swimming, and PFS curves were plotted using GraphPad Prism 10.0.2 software.

## 3 Results

### 3.1 Patient characteristics

Nineteen patients with a median age of 64 years (range, 50-70) were enrolled between December 2020 and February 2023. The baseline patient characteristics are summarized in **Table 1**. Among them, 16 (84.21%), 2 (10.53%), and 1 (5.26%) presented with stage IV, III, and II disease, respectively (according to the 8th edition of the American Joint Committee on Cancer staging system). All 19 patients underwent three cycles of induction chemotherapy with N-P + DDP/NDP + S-1. Subsequently, seven patients underwent surgery, whereas four, two, and two patients received radiotherapy, chemotherapy, and chemotherapy plus immune checkpoint inhibitors (ICIs). Additionally, four patients chose to discontinue treatment after understanding the consequences. The median follow-up time was 17.43 months (range, 5-32).

**Table 1:** Baseline demographic and disease characteristics.

Characteristics	Number of patients (%) N=19
Age, years	64 (50–70)
Sex	
Male	19 (100)
Female	0
T Stage	
T1	2 (10.5)
T2	6 (32.0)
T3	2 (10.5)
T4a	4 (21.0)
T4b	5 (26.0)
N Stage	
N0	2 (10.5)
N1	3 (16.0)
N2a	1 (5.0)
N2b	6 (32.0)
N2c	2 (10.5)
N3b	5 (26.0)
Summary Stage	
IVB	8 (42.0)
IVA	8 (42.0)
III	2 (11.0)
II	1 (5.0)
Differentiation degree	
Poor	4 (21.0)
Moderate	9 (47.0)
High	3 (16.0)
Unknown	3 (16.0)
ECOG performance status	
0	17 (89.5)
1	2 (10.5)

ECOG, Eastern Cooperative Oncology Group.

### 3.2 Efficacy

**Figure 2:**
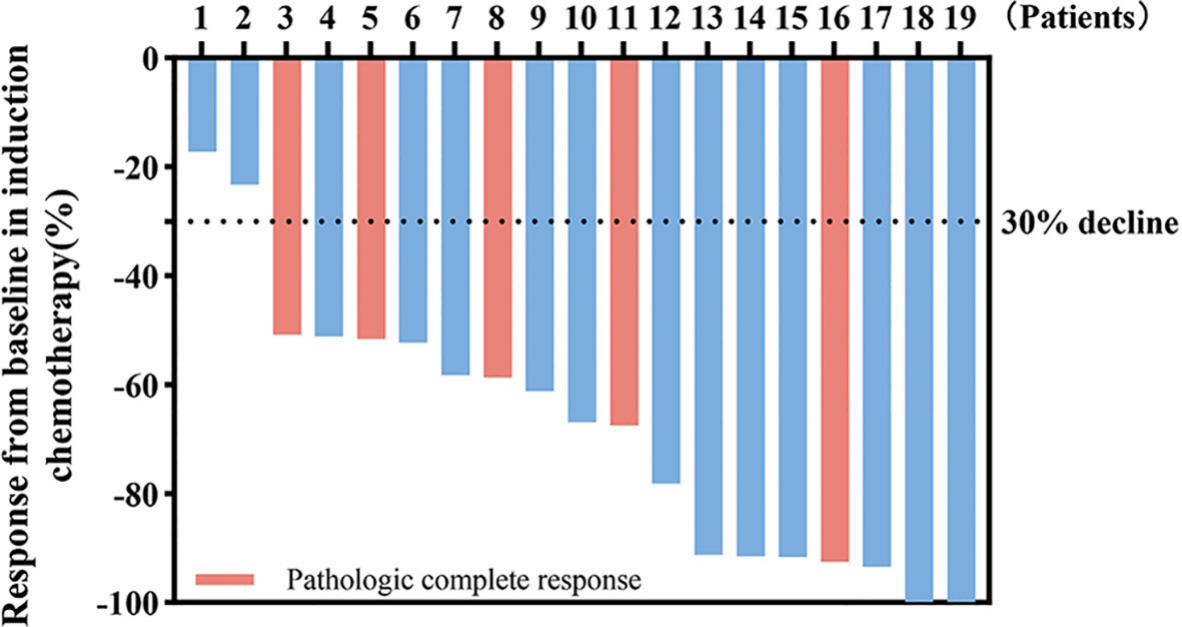
Waterfall plot of percentage change in tumor response compared with that at the baseline. Color indicates the type of response. The dashed line at -30% represents the boundary for determining the partial response.

**Figure 3:**
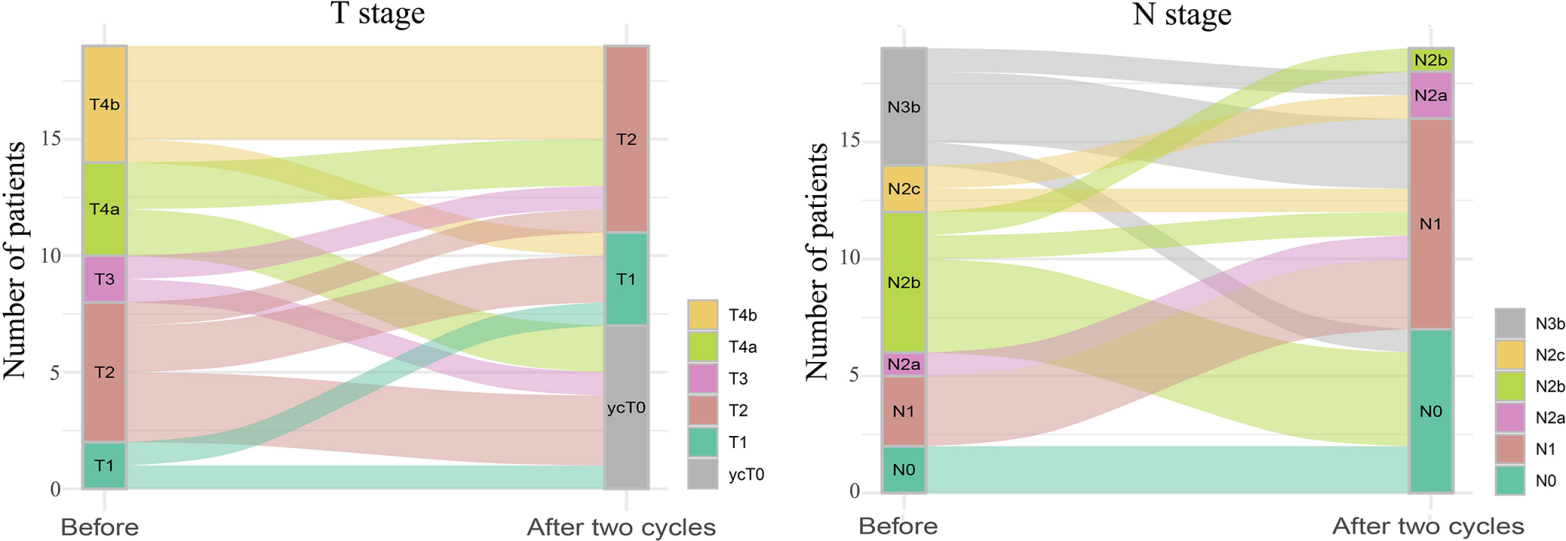
Sankey diagram of tumor (T) and lymph node (N) staging changes before and after two cycles of induction chemotherapy for hypopharyngeal cancer. The size of the line represents the number of people in the T or N stages.

**Figure 4:**
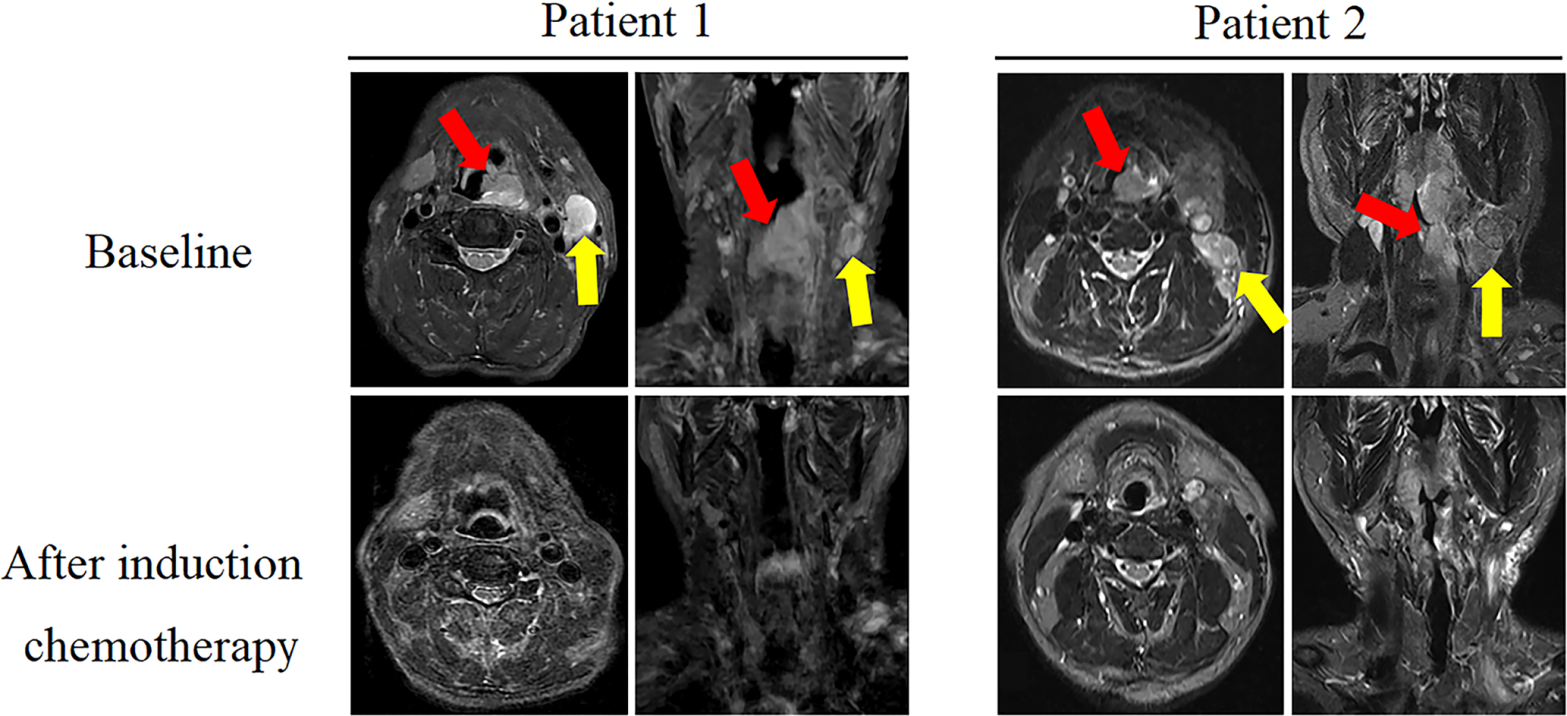
Comparison of magnetic resonance imaging (MRI) images (axial and coronal T2WI images) between baseline and after two cycles of induction chemotherapy in two typical cases. In the baseline image, red and yellow arrows indicate primary lesion and lymph node metastasis, respectively. After induction therapy, the corresponding area of the lesion disappeared.

All 19 patients completed three cycles of induction chemotherapy. **Figure 2** depicts the percent changes in the sum of the diameters of the target lesions compared with that at baseline after the second cycle of chemotherapy. Among these patients, 2 (10.53%), 15 (78.94%), and 2 (10.53%) exhibited radiological complete response, partial response, and stable disease, respectively. The ORR and DCR were 89.47% and 100%, respectively. A decreasing trend in T and N staging was observed after the second cycle of induction chemotherapy compared with that at baseline in these patients (**Figure 3**). **Figure 4** illustrates the typical imaging findings before and after chemotherapy. Following chemotherapy, 94.74% (18/19) of patients met the criteria for radical surgery according to the otolaryngologist’s evaluation.

**Figure 5:**
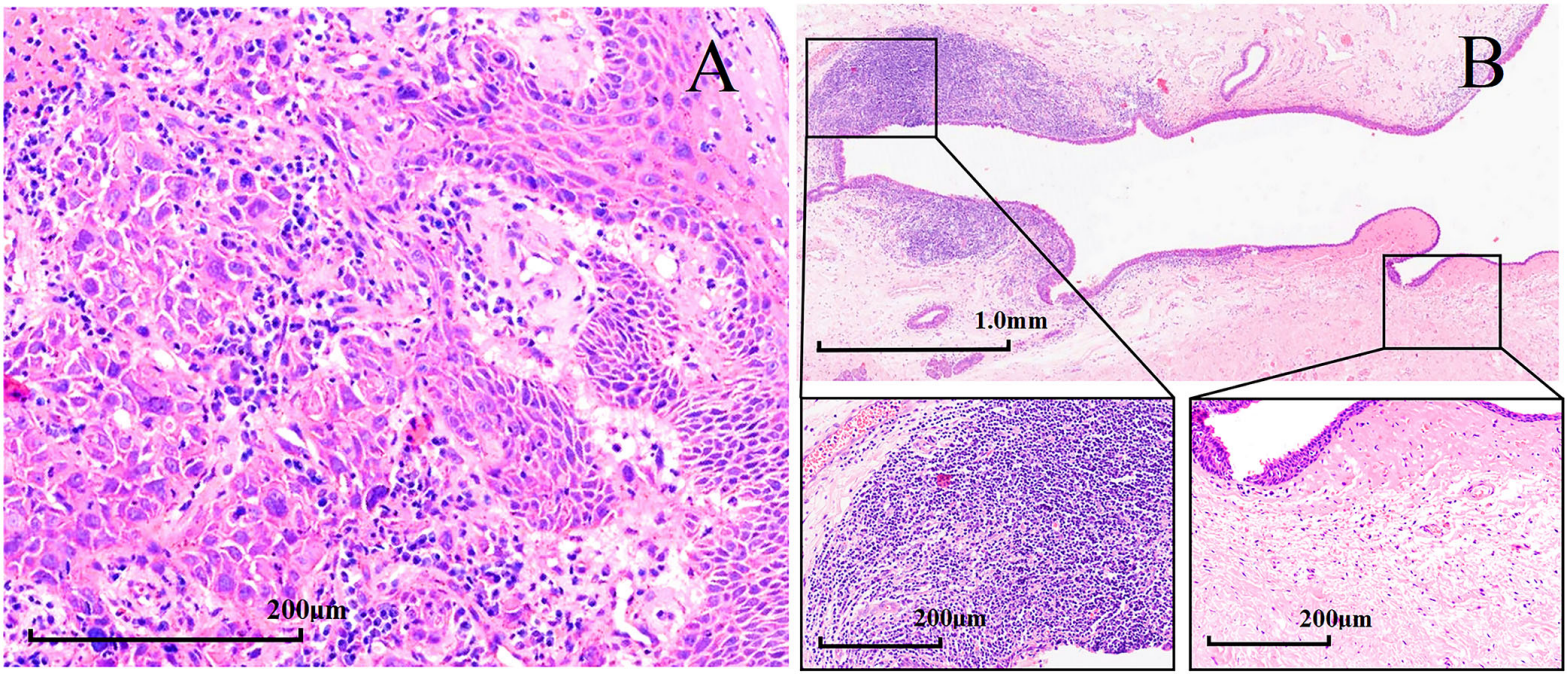
Hematoxylin and eosin (H&E) staining in patients with hypopharyngeal squamous cell carcinoma with pathological complete response (pCR, magnification × 200). H&E staining **(A)** before induction chemotherapy and **(B)** after surgery.

After being informed of the advantages and disadvantages of subsequent treatments, patients independently selected their preferred treatment plan after induction chemotherapy. Seven patients underwent surgery after three cycles, including two who received postoperative radiation therapy. Among the patients who underwent surgery, three patients underwent subtotal laryngectomy, another three patients underwent total laryngectomy, and one patient had incomplete information. Among the seven surgical patients, six underwent tracheotomy, four underwent tracheostomy, and one patient had incomplete information. The pathological complete response rate (pCR) was 71.43% (5/7), according to the pathological evaluation of the postoperative resection samples. **Figure 5** shows the typical pathological changes in patients with a pCR before and after induction chemotherapy.

**Figure 6:**
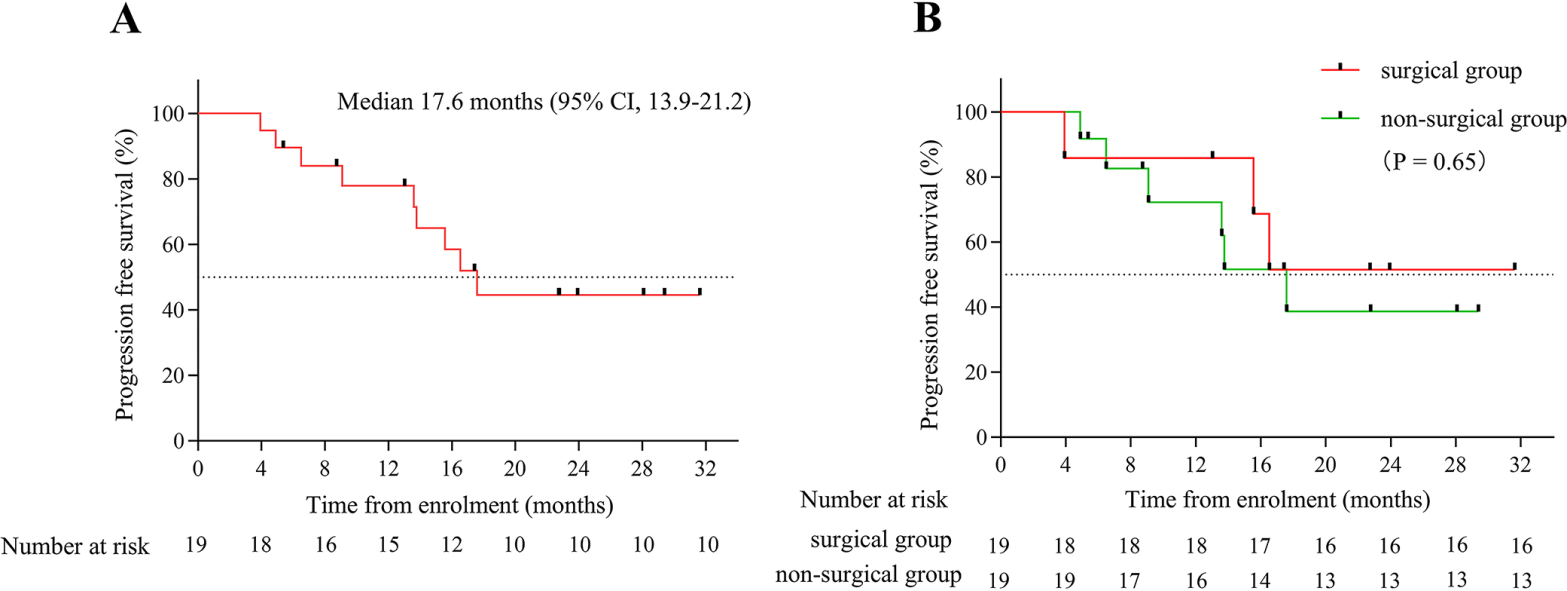
**(A)** Kaplan–Meier plots for progression-free survival (PFS) in the study population of 19 patients. **(B)** Kaplan–Meier plots for PFS of surgical and non-surgical groups.

By the end of August 2023, the median follow-up time was 17.43 months, and the median PFS (mPFS) was 17.6 months (95% CI, 13.9-21.2) (**Figure 6A**). The mPFS of patients who underwent surgery was uncertain, whereas that of patients who did not undergo surgery was 17.6 months, with no significant difference between the groups (*p*=0.65, **Figure 6B**). However, patients with surgically confirmed pCR exhibited a longer mPFS than those in non-surgical subgroups (17.6 vs. 24.08 months), there was no significant difference between the groups (*p*=0.49). At the end of the study, one patient died, whereas all others survived (18/19, 94.73%).

**Figure 7:**
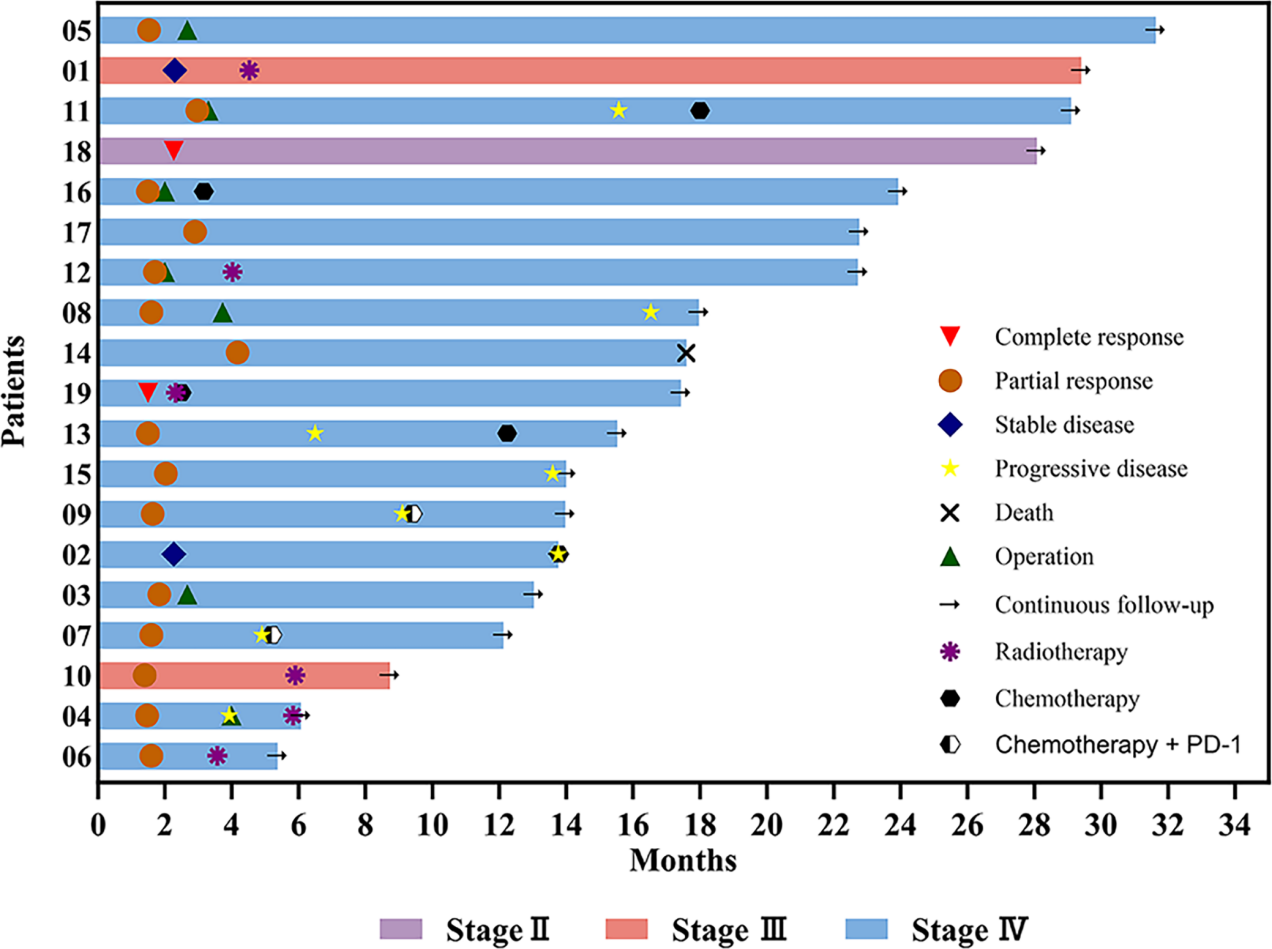
Swimming plot of the clinical process of patients with hypopharyngeal cancer receiving induction chemotherapy.

Eight patients experienced disease progression (8/19, 42.11%). Three patients who underwent surgery exhibited disease progression (3/7, 42.86%), including two patients with pCR (2/5, 40%). Disease progression occurred in five patients who did not undergo surgery (5/12, 41.67%). Among the four patients who did not receive other treatments after induction chemotherapy, two (2/4, 50%) remained in sustained remission, with only one patient showing disease progression. The four patients who received radiotherapy after induction chemotherapy did not exhibit disease progression. **Figure 7** illustrates the diverse treatments administered and the efficacy evaluations during the study period.

### 3.3 Safety

**Table 2** summarizes the incidences of treatment-related AEs (TRAEs) during the study period. The most common AEs (≥ grade 1) included anemia, leukemia reduction, alopecia, fatigue, and thrombocytopenia. Grade 3 treatment-emergent AEs (TEAEs) were predominantly alopecia, leukopenia, and anemia. No grade 4/5 TEAEs were observed. Supportive treatment effectively helped to control almost all AEs. One patient died during the COVID-19 pandemic in January 2023. The investigators concluded that this death was unrelated to the primary tumor or TRAEs.

**Table 2:** Adverse events.

Treatment-related adverse events	N-P plus cisplatin plus S-1 (n = 5)		N-P plus nedaplatin plus S-1 (n = 14)		All Grades, No. (%) (n =19)
	Total, No. (%)	Grade 3, No. (%)	Total, No. (%)	Grade 3, No. (%)	
Anemia	5 (100)	1 (20)	9 (64.3)	2 (14.3)	14 (73.7)
Thrombocytopenia	1 (20)	-	4 (28.6)	-	5 (26.3)
Leukemia Reduction	5 (100)	2 (40)	7 (50)	3 (21.4)	12 (63.2)
Fever	3 (60)	-	-	-	3 (15.8)
Pain	-	-	2 (14.3)	-	2 (10.5)
Nausea	1 (20)	-	1 (7.1)	-	2 (10.5)
Decreased appetite	1 (20)	-	2 (14.3)	-	3 (15.8)
Skin erythema	1 (20)	-	-	-	1 (5.3)
Alopecia	3 (60)	3 (60)	6 (42.9)	4 (28.6)	9 (47.4)
Dizziness	2 (40)	-	2 (14.3)	-	4 (21.1)
Numbness of the limbs	1 (20)	-	3 (21.4)	-	4 (21.1)
Fatigue	2 (40)	-	5 (35.7)	-	7 (36.8)
Hearing loss	-	-	1 (7.1)	-	1 (5.3)

## 4 Discussion

In this study, we analyzed the efficacy and safety of the N-P+DDP/NDP+S-1 combination therapy in patients with hypopharyngeal cancer. To our knowledge, this is the first study to use N-P+ DDP/NDP +S-1 for the treatment of hypopharyngeal cancer. After two cycles of induction chemotherapy, the patients exhibited improved short-term efficacy and safety. The ORR was 89.47%, and all treatment-related AEs were controlled with symptomatic treatment.

Among head and neck tumors, hypopharyngeal tumors exhibit the worst prognosis, primarily owing to their tendency to be identified at an advanced stage, posing challenges for early diagnosis (15). Currently, a combination of surgery, chemotherapy, and radiation therapy has shown favorable efficacy for locally advanced hypopharyngeal cancer (16, 17). Several studies have shown that the TPF regimen induction chemotherapy improves the survival rate and larynx preservation in patients with unresectable hypopharyngeal cancer (12, 18). Consequently, the NCCN recommends the incorporation of TPF to the induction chemotherapy regimen (7). The European Organization for Research and Treatment of Cancer 24891 (EORTC24891) study (10) included patients with resectable squamous cell carcinoma of the larynx who were randomly assigned to receive PF regimen induction chemotherapy or total laryngectomy. The median overall survival (OS) and mPFS were 3.67 and 2.1 years, respectively, in the chemotherapy arm and 2.1 and 1.6 years, respectively, in the surgery arm. There was no difference in 10 year OS between upfront surgery vs induction chemotherapy in EORTC24891 study (10). The standard TPF regimen was evaluated in patients with hypopharyngeal tumors in the GORTEC 2000-01 study (11), the overall response rate after induction chemotherapy in the TPF and PF groups were 80.0% (41.8% complete response and 38.2% partial response) and 59.2% (30.1% complete response and 29.1% partial response) (*p*=0.002), respectively. OS and disease-free survival were not significantly improved in the TPF arm versus the PF arm (respectively, *p*=0.28, *p*=0.21) (11). Kim et al. evaluated the efficacy and safety of induction chemotherapy with docetaxel combined with S-1 and radiotherapy with concurrent daily cisplatin in locally advanced head and neck carcinoma (19). They found that the ORR after induction chemotherapy was 64% (32/50), however, after completion of induction chemotherapy, 40 patients received concurrent chemoradiotherapy, and the ORR was 100% (19). Our study showed that the ORR was similar to the TPF regimen. The ORR in our study was significantly higher than that of the docetaxel and S-1 combined induction regimen, which may be because our induction regimen contained platinum, whereas there was no platinum in the induction chemotherapy regimen studied by Kim et al.

Sittitrai et al. found that the tumor resection rate after TPF-induced chemotherapy for unresectable stage IVb laryngeal and hypopharyngeal cancers was 78.6% (20). In our study, otolaryngologists confirmed that patients with stage IVb disease met the criteria for radical surgical treatment after induction chemotherapy, suggesting that this combination regimen may be a potent induction therapy for TPF. Previous research has shown that the median OS of the resectable and unresectable groups was 20.0 months (16.0-35.5) and 9.5 months (6.0-15.0), respectively (*p*=0.008) (20). At the end of follow-up, no patient died of primary tumor and TRAEs in our study, and median OS was not observed. Moreover, mPFS was not observed in patients who underwent surgery after induction chemotherapy, and the mPFS in patients who did not undergo surgery was 17.6 months, there was no significant difference between the groups (*p*=0.65). Some patients achieved pCR after surgery in our study, and further investigation is warranted to confirm whether achieving pCR after induction chemotherapy can reliably help predict improved survival benefits.

A study of chemotherapy combined with radiotherapy (I) versus surgery and postoperative radiotherapy (II) for advanced hypopharyngeal cancer showed that 9 of 30 patients (30%) in group II developed a local recurrence, compared to 11 of 26 patients (42%) in group I (*p*=0.57) (21). A study conducted by Sittitrai et al. on unresectable stage IVb laryngeal cancer and hypopharyngeal cancer found that locoregional recurrence rate in the resectable and unresectable groups was 44.4% and 85.7%, respectively (20). In our study, eight patients experienced disease progression or recurrence, and recurrence rates were similar between patients who underwent surgery and those who did not (42.86% vs 41.67%). We speculate that this may be due to the small sample, different experimental designs, different doses and times of chemotherapy, and other factors.

Definitive radiotherapy alone or partial laryngopharyngectomy is a treatment option for early-stage squamous cell carcinoma of the hypopharynx (22, 23). At present, there are no studies on the use of induction chemotherapy for early hypopharyngeal cancer. One patient in our study had stage II disease and achieved a complete response after induction chemotherapy. Therefore, we speculate that specific subtypes of patients with hypopharyngeal cancer might achieve an ideal disease prognosis with chemotherapy alone. Induction chemotherapy may be a viable option for improving laryngeal protection in patients with early-stage disease. However, further research is required for validation.

Instead of the standard TPF regimen, we chose a platinum-based combination of N-P and S-1. S-1 is a compound preparation consisting of tegafur, oteracil, and gimeracil (24). Tegafur is the primary active ingredient of S-1, which is converted into 5-fluorouracil (5-FU) in the body. Gimeracil inhibits dihydropyrimidine dehydrogenase and prevents the breakdown of 5-FU, thereby prolonging its residence time in the body and enhancing the drug’s efficacy. Oteracil reduces the activity of 5-FU in normal intestinal tissues by blocking its phosphorylation, thereby reducing the damage to normal tissues and minimizing the occurrence of adverse events (25–27). S-1 was associated with a lower rate of febrile neutropenia, toxicity-related deaths, grade 3-4 stomatitis, and mucositis than 5-fluorouracil (28). S-1, an oral medication, may help prevent AEs associated with intravenous injections. S-1 has been approved for the treatment of gastric cancer, colon cancer, rectal cancer, pancreatic cancer, non-small cell lung cancer, head and neck cancers and so on. N-P is an albumin-bound, solvent-free form of paclitaxel (29). Compared with docetaxel, N-P increases the incidence of peripheral sensory neuropathy but reduces that of grade 3-4 neutropenia and febrile neutropenia (30, 31). Grade 3-4 late toxicities of the larynx in the TPF arm were fewer than those in the PF arm (9.3% vs 17.1%), and three patients in the TPF arm died of acute toxicity of chemotherapy (11). Mucous membrane, salivary gland, larynx, and subcutaneous tissue toxicities were the most common AEs (11). In our study, phlebitis, febrile neutropenia, or anaphylaxis was not observed, and no grade 4-5 AEs were observed with the combination of N-P + DDP/NDP + S-1 in the induction phase. No AEs such as stomatitis and esophagitis were noted, which may be related to the use of S-1. None of the patients died of TRAEs. A study of first-line N-P in patients with advanced ovarian cancer and a study of N-P monotherapy for relapsed small-cell lung cancer showed that the incidence of alopecia and limb numbness increased (32, 33). Compared with that in previous studies (11), the incidence of alopecia and limb numbness increased in our study, potentially owing to N-P. Furthermore, we found that the incidence of adverse events was significantly higher among patients who received cisplatin - based induction chemotherapy regimens compared to those who were administered nedaplatin - based regimens. These AEs resolved after symptomatic treatment, and none of the patients discontinued treatment because of chemotherapy-related AEs. Thus, the safety of the induction regimen we studied is manageable.

Despite these encouraging results, our study had some inherent limitations. The retrospective nature of the study introduced biases, and the absence of a control group made it challenging to definitively conclude the superiority of the N-P + DDP/NDP + S-1 combination over TPF. Moreover, the small number of patients and relatively short follow-up period may not fully capture long-term outcomes. Therefore, a rigorously designed multicenter randomized controlled study is still required to further validate the efficacy and safety of the combination of N-P + DDP/NDP + S-1 in the induction treatment of hypopharyngeal cancer.

In conclusion, the N-P + DDP/NDP + S-1 combination demonstrated a favorable safety profile during the induction phase of hypopharyngeal cancer therapy. Additionally, we speculate that chemotherapy alone can yield long-term benefits in some patients with hypopharyngeal cancers. Future studies should prioritize prospective clinical trials to further investigate the potential benefits of this regimen.

## Data Availability

The data analyzed in this study is subject to the following licenses/restrictions: The dataset can be obtained with the approval of the Affiliated Hospital of the Guilin Medical University. The data that support the findings of this study are available from the corresponding author, FX, xuefeng_doctor@126.com, on reasonable request.
